# Bridging the Gap in Early Cerebral Palsy Detection: Primary Care Providers' and Specialists' Perspectives on Implementing PROMPTs for Referral

**DOI:** 10.1111/cch.70287

**Published:** 2026-05-10

**Authors:** Annette Majnemer, Darcy Fehlings, Martina Alkot, Mae Ru Sanford, Tatiana Ogourtsova

**Affiliations:** ^1^ Faculty of Medicine and Health Sciences, School of Physical and Occupational Therapy McGill University Montreal Quebec Canada; ^2^ Research Institute of the McGill University Health Center Montreal Quebec Canada; ^3^ Holland Bloorview Children's Rehabilitation Hospital Toronto Ontario Canada; ^4^ Department of Paediatrics University of Toronto Toronto Ontario Canada; ^5^ RESI‐ALLIANT KID Laboratory Research Center of the Jewish Rehabilitation Hospital, Integrated Health and Social Services Center of Laval Laval Quebec Canada; ^6^ Centre for Interdisciplinary Research in Rehabilitation of Greater Montreal Montreal Quebec Canada

**Keywords:** cerebral palsy, early detection, focus groups, interviews, primary care, qualitative, referral tool, specialists

## Abstract

**Background:**

Cerebral palsy (CP) is the most common childhood‐onset physical disability. Although diagnosis can be made within the first year of life, many children, particularly those with milder presentations, are diagnosed well after their second birthday. Delayed recognition limits access to early interventions and increases caregiver stress. The Early Detection and Intervention Toolkit for CP (EDIT‐CP) was developed to bridge this gap, including PROMPTs for referral (Primary‐care Referral of Motor‐impaired children: Physician Tools) to guide early identification in primary care.

**Objectives:**

This study aimed to explore how PROMPTs can be optimized and implemented across Canadian healthcare contexts. Specifically, we explored (1) primary care providers' perspectives on PROMPTs' feasibility, usability and integration into well‐baby visits and surveillance tools; and (2) paediatric specialists' perspectives on referral patterns, age of diagnosis, barriers to early detection and system‐level dissemination strategies.

**Methods:**

In a qualitative study design, participants were first provided access to EDIT‐CP and PROMPTs for referral for review prior to data collection. Semi‐structured interviews were then conducted with primary care providers (*n* = 11), and a focus group (*n* = 7) and interviews with paediatric specialists (*n* = 2) to explore their perspectives on feasibility, usability, referral patterns and system‐level implementation. Data were analysed using a hybrid inductive–deductive approach.

**Results:**

Primary care providers highlighted the importance of clearer referral thresholds and pathways, with PROMPTs perceived as a useful facilitator. Specialists highlighted progress in earlier diagnosis for infants with high probability of CP, ongoing hesitancy to diagnose before age two and inequities in access. Both groups valued embedding PROMPTs for referral into existing workflows, emphasized the need for training and dissemination strategies and underscored the importance of communication with families.

**Contribution:**

Findings show strong alignment among providers on the need for structured, practical and scalable early detection pathways. PROMPTs for referral extend early detection into frontline practice, offering a strategy to reduce diagnostic delays and promote equitable access to early intervention for children with CP.

## Summary

- Early recognition of cerebral palsy (CP) in primary care is feasible and can be strengthened by embedding concise, observable red flags into existing well‐baby surveillance tools.
- PROMPTs for referral extend early detection capacity beyond tertiary centres by equipping frontline clinicians with practical, actionable indicators that support timely referral pathways for infants with subtle or mild motor delays.
- Primary care providers and paediatric specialists strongly agree on the need for clearer, structured referral pathways and consistent communication strategies, including the use of ‘high probability of CP’ to support earlier diagnosis and family‐centred care.
- System‐level supports such as EMR (Electronic Medical Record) integration, CME (Continuing Medical Education)‐accredited training and targeted dissemination to family physicians, nurse practitioners and trainees are essential to achieving scalable, equitable implementation of early detection practices across Canada.
- Reducing diagnostic delays, particularly for children without classic neonatal risk factors, represents a public health and health equity priority; optimizing PROMPTs and embedding them within routine workflows can meaningfully improve access to early intervention.

## 1 Introduction

Cerebral palsy (CP) refers to a group of permanent disorders of the development of movement and posture, causing activity limitation, that are attributed to nonprogressive disturbances that occurred in the developing foetal or infant brain (Rosenbaum et al. 2007). It is the most common childhood‐onset physical disability worldwide, affecting approximately 2.2 per 1000 children in Canada—a rate projected to increase to 2.4 per 1000 by 2031 (Amankwah et al. 2020). CP has lifelong implications not only for motor function and health outcomes but also for participation, education, employment and quality of life. The socioeconomic burden is substantial: Canadian data estimate that direct healthcare costs for children aged 1–4 years with CP average over $11 700 annually, compared to $600 for children without CP (Amankwah et al. 2020). These costs increase with motor severity and are compounded by caregiver burden, lost productivity and the need for long‐term supports. Early detection creates opportunities for timely intervention during critical periods of neuroplasticity, which can improve functional outcomes, reduce secondary complications and better support families (Novak et al. 2017; Spittle and Morgan 2025). Given its lifelong impact and substantial social and economic costs, investing in earlier detection and intervention for CP is not only a clinical imperative, it is a public health and health equity priority.

The clinical features of CP, particularly motor dysfunction, are known to emerge gradually as developmental milestones are missed or determined as being atypical. This makes timely detection challenging, especially in community and primary care settings. Tools like the General Movements Assessment (GMA), Hammersmith Infant Neurological Examination (HINE) and neuroimaging enable accurate diagnosis within the first 6–12 months of life in infants with high probability of CP (Novak et al. 2017). These assessments also permit early prognostication of motor severity, including determination of Gross Motor Function Classification System (GMFCS) level within the first year of life. During the fidgety movement period (3–5 months postterm), absent fidgety movements and low Motor Optimality Score–Revised scores (e.g., < 8) are strongly associated with later severe motor impairment (GMFCS III–V), including a high probability of nonambulatory outcomes (Paris et al. 2023). Similarly, low HINE scores in early infancy (e.g., < 40 at 3–6 months corrected age) are highly predictive of GMFCS III–V and nonambulatory trajectories (Novak et al. 2017). This early prognostic capability is a cornerstone of modern intervention planning, as it enables clinicians to identify, with a high degree of accuracy, children who will likely require specialized mobility supports and proactive orthopaedic monitoring long before independent ambulation would otherwise be expected. However, the use of these tools remains limited in Canadian practice, particularly outside of tertiary centres with neonatal intensive care units and follow‐up. Nationally, the average age of CP diagnosis remains between 12 and 24 months, and many children with milder impairments are diagnosed well after their second birthday (Boychuck et al. 2019; Boychuck et al. 2020c). A recent retrospective review of the Canadian Cerebral Palsy Registry determined that in British Columbia, children classified as GMFCS Levels I–II (i.e., who make up the majority of the CP population) were significantly more likely to receive a diagnosis after 24 months of age compared to peers with more severe motor impairments (McIntosh et al. 2025). Importantly, these diagnostic delays are not unique to British Columbia. A national environmental scan revealed significant variability in referral practices across Canada, with inconsistent use of standardized tools and unclear pathways to diagnosis and intervention for children with suspected CP (Boychuck et al. 2020c). The study highlighted that many children—particularly those with milder motor impairments—are referred late or not at all, and that primary care providers often lack guidance on how and when to initiate referrals. These findings underscore a systemic need for clear, evidence‐based pathways that equip clinicians across the continuum of care to recognize early signs of CP, initiate timely referrals and engage families in coordinated intervention planning.

Furthermore, delayed diagnosis limits access to early, neurodevelopmentally oriented interventions and increases caregiver stress. Studies have shown that many caregivers felt their child's diagnosis could have been delivered sooner and described having to persistently advocate to be taken seriously, especially when motor signs were subtle (Williams et al. 2021). Others report that ambiguous or late diagnostic disclosure heightened their anxiety and undermined trust in the healthcare system (Baird et al. 2000; Dagenais et al. 2006). One important driver of these delays is provider uncertainty. Many primary care providers report feeling underprepared to recognize early markers of CP, contributing to missed opportunities for earlier diagnosis and referral (Scherzer et al. 2012). This is especially problematic for infants who present with mild or non‐specific signs and lack classic risk factors.

The **E**arly **D**etection and **I**ntervention **T**ools—**C**erebral **P**alsy (**EDIT‐CP**) was developed to bridge the evidence‐to‐practice gap by promoting evidence‐based care and empowering families and clinicians through accessible, codesigned resources. It comprises two main components: **PROMPTs** for referrals (**P**rimary‐care **R**eferral **o**f **M**otor‐impaired children: **P**hysician **T**ool**s**), which helps clinicians recognize early indicators of CP in infants and toddlers; and an **Early Intervention Platform** (Hanson et al. 2024) that translates best available evidence into more than 20 user‐friendly learning modules for rehabilitation professionals and caregivers.

**FIGURE 1:**
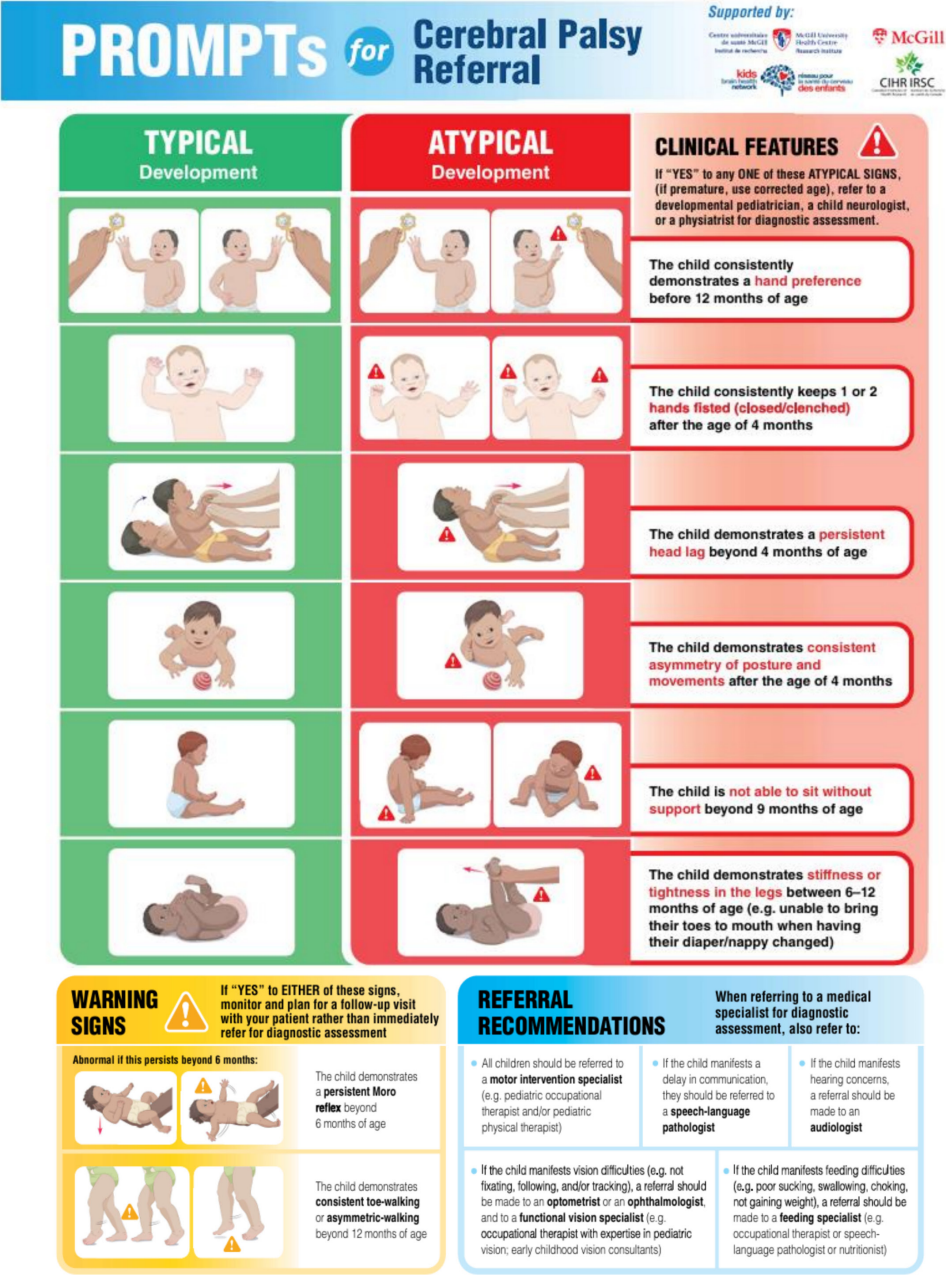
PROMPs for Referral.

More specifically, over the past decade, extensive work by Canadian researchers and numerous international collaborations has identified practical, observable early signs that can assist primary care physicians in determining which infants may require further assessment (Boychuck, Andersen, Bussières, et al. 2020). These indicators are embedded in the **PROMPTs for referrals** (Figure 1), along with guidance for timely and coordinated referrals to medical and rehabilitation specialists. Embedding these resources into routine primary care workflows is expected to promote early CP recognition as well as timely intervention. However, to support sustainable adoption of this tool across diverse healthcare settings, it is essential to understand how it is perceived, used and integrated by healthcare providers in real‐world practice.

Building on this foundational work, the overarching aim of this study was to explore how the **EDIT‐CP PROMPTs for referral** can be optimized and implemented across the continuum of care, including primary care, community paediatrics and tertiary developmental and rehabilitation services in Canada to support earlier recognition and intervention for children with CP. The specific objectives were to (1) explore the perspectives of primary care providers on the feasibility and usability of the clinical **PROMPTs for referral**, their integration into well‐baby visits and existing surveillance tools and strategies to support adoption in frontline practice; and (2) Explore specialists' perspectives on current referral patterns, variability in age at CP diagnosis and barriers to early identification, in order to contextualize system‐level factors that may influence the dissemination and integration of PROMPTs and other early detection resources across healthcare settings.

## 2 Methods

### 2.1 Study Design

This study used a qualitative study design in the form of semi‐structured interviews and focus group discussions. Ethics approval for this study was obtained from the Research Institute of the McGill University Health Center review board (Approval No. MP‐37‐2023‐8827, Date of Approval: 2023‐04‐20). All participants provided written informed consent prior to participation.

### 2.2 Study Procedures

This study comprised two components conducted between March and August 2024. First, online semi‐structured interviews were conducted with primary care providers by a fully bilingual study coordinator. These interviews focused on the feasibility and usability of integrating **PROMPTs for referral** into the well‐baby care context. Once consent was obtained, participants were provided with a preinterview package (see Data S1) and invited to review the relevant materials in advance, including the Rourke Baby Record, the ABCDaire and the **PROMPTs referral/EDIT‐CP** tools. Participants were given approximately 1 week between providing consent and attending the interview to allow sufficient time for review. Interview questions probed views on ease of access and navigation, clarity of graphics and content, likelihood of integration into routine practice and existing tools, potential barriers and facilitators to use and recommendations for refinement and dissemination. Interviews were conducted in either English or French, based on participant preference.

In the second component, consultations were carried out with paediatric specialists. A focus group was held with developmental paediatricians and child neurologists from across Canada, supplemented by individual online interviews with additional specialists. The focus groups were facilitated by authors (AM and TO) and the interviews were conducted by a research assistant. Specialists were asked to reflect on observed changes in referral age, referral sources and types of CP identified earlier and to provide input on barriers to earlier diagnosis and strategies for broader dissemination of early detection tools to primary care and community providers.

For both components, sessions were conducted via Zoom, audio‐recorded with permission and transcribed verbatim. The interview and focus group guide are outlined in Supplementary file Data S1.

### 2.3 Study Participants

Participants were eligible for inclusion if they were healthcare providers involved in the care of infants and young children in Canada and had a role in developmental surveillance, referral or diagnosis of CP. For Component 1, this included primary care providers (family physicians, general paediatricians, nurse practitioners and nurse clinicians) engaged in well‐baby care and monitoring early developmental milestones. For Component 2, inclusion criteria targeted paediatric specialists (developmental paediatricians, paediatric physiatrists and paediatric neurologists) working in tertiary care or academic hospital settings with expertise in CP diagnosis and management. For Component 1, we used bilingual recruitment materials and targeted Francophone and Anglophone primary care providers across rural, suburban and urban settings; for Component 2, recruitment extended across multiple provinces to ensure diverse regional representation.

### 2.4 Analysis

A hybrid inductive–deductive thematic analysis was used to analyse data from both study components (Fereday and Muir‐Cochrane 2006). All interviews and focus groups were deidentified before analysis. An initial coding framework was developed based on the interview and discussion guides, which reflected the main areas of inquiry across both components. This framework provided an organizing structure for coding while allowing new and unexpected ideas to emerge through line‐by‐line inductive coding. Coding and theme development were conducted iteratively, with regular team discussions to refine definitions and ensure consistency. Each transcript was reviewed independently by at least two members of the research team (TO and MA), and discrepancies were resolved by consensus. French‐language interviews were analysed in their original language, as both members of the analytic team are fully bilingual (TO and MA). NVivo software (QSR International) was used to support data management and analysis. To enhance transparency and rigour, the frequency of participants mentioning each theme was recorded and expressed as number (*n*) and proportion (%) of participants within each component. These counts do not imply statistical generalizability but provide an indication of the relative salience of themes within the sample. Representative quotes were retained in the analysis to illustrate key findings, whereas convergence and divergence across primary care and specialist perspectives were narratively compared, where appropriate.

## 3 Results

### 3.1 Study Participants

The demographic characteristics of study participants are reported in Table 1. In total, twenty (*n* = 20) healthcare providers participated. Component 1 included 11 (*n* = 11) primary care clinicians: family physicians, paediatricians, nurse practitioners and a nurse clinician, representing varied levels of experience, language and practice settings. Component 2 included nine paediatric specialists, all based in urban tertiary care centres and working exclusively with paediatric populations.

**TABLE 1:** Characteristics of study participants.

Characteristic	Component 1: Primary care providers (*n* = 11)	Component 2: Paediatric specialists (*n* = 9)
Profession *n* (%)	Family physicians: 5 (46) General paediatricians: 2 (18) Nurse practitioners: 3 (27) Nurse clinician: 1 (9)	Developmental paediatricians: 6 (66) Paediatric neurologists: 2 (22) Paediatric physiatrist: 1 (11)
Gender	Female: 9 (82)	Female: 4 (44)
*n* (%)	Male: 2 (18)	Male: 5 (55)
Years of clinical experience *n* (%)	1–5 years: 4 (36) 6–10 years: 2 (18) 11–15 years: 3 (27) 16–25 years: 2 (18)	1–5 years: 1 (11) 6–10 years: 2 (22) 11–15 years: 2 (22) 16–25 + years: 4 (44)
Paediatric population served *n* (%)	10%–20% under 18: 2 (18) 30%–50% under 18: 4 (36) 100% under 18: 5 (46)	100% under 18: 9 (100)
Area of employment *n* (%)	Rural: 2 (18) Urban: 8 (73) Suburban: 1 (9)	Urban: 9 (100)
Province	Quebec: 11 (100)	Ontario: 4 (44) Quebec: 2 (22) Alberta: 2 (22) British Columbia: 1 (11)

### 3.2 Component 1—Primary Care Providers—Perspectives on PROMPTs for Referral

Semi‐structured interviews with primary care providers (*n* = 11) yielded six major themes concerning the feasibility, usability and integration of early detection attributes for CP into well‐baby care.

#### 3.2.1 Theme 1. Feasibility of Integrating Early Detection PROMPT Attributes Into Well‐Baby Care

Most participants (*n* = 9/11, 82%) emphasized the need for clearer referral pathways and milestone interpretation. Providers suggested guidance on when a child should be referred to a paediatrician versus a subspecialist, in order to ensure timely care without overburdening neurologists. As one general paediatrician explained:

There is a role … if a family doctor sees these red flags to refer to a pediatrician for further evaluation. […] Neurologists end up becoming very like third or fourth line in the reality of resources. (P6_Pediatrician)

However, the feasibility of this tiered referral approach is dependent on local healthcare context, particularly the availability, scope of practice and gatekeeping role of community paediatricians. In some regions, community paediatricians function as primary consultants for developmental concerns, whereas in others, children may be referred directly to subspecialists due to workforce distribution, access constraints or funding structures.

Clinicians also highlighted the importance of extending milestone ranges to reduce false positives and consistently adjusting for corrected age in premature infants.

Simply put a mention … not to forget to do the calculation of the corrected age. Maybe in the introduction: “Deduct the number of weeks if the child is premature.” (P10_Nurse Clinician)

#### 3.2.2 Theme 2. Experiences With Existing Surveillance Tools and Their Integration of PROMPT (Rourke Babe Record, ABCDaire)

Seven participants (*n* = 7/11, 64%) reflected on their use of surveillance tools. The Québec‐based ABCDaire was generally preferred for usability, space for notes and French availability, whereas the Rourke Baby Record was considered more detailed but overly condensed and less user‐friendly. Regardless of the tool, providers stressed the need to embed clearer CP‐specific red flags to support earlier recognition:

I always felt the ABCDaire was more user‐friendly … there were places to write. The Rourke is too condensed. (P5_Family Medicine/Peds Urban)

I found in the Rourke, in terms of explanations and advice, it was more complete than the ABCDaire. The physical exam is more complete in the ABCDaire, however, and there is more room to write at the bottom. (P10_Nurse Clinician, translated)

Ours [ABCDaire] is integrated into our EMR [electronic medical records]. And there is a section of red flags. […] It's very obvious. The way it's formulated is like checkboxes we can actually tick off as we go through it. (P6_GP)

We all use the Rourke, and we'll all go down to the bottom to look at the information aspect. So, if there's a way to link the graphic and relevant information, that would be great. (P4_Family Medicine Rural)

#### 3.2.3 Theme 3. Usability of the EDIT‐CP Toolkit and Web‐Resources

Seven participants (*n* = 7/11, 64%) found the website to be a helpful bilingual resource but noted both strengths and areas for improvement. Although many valued its clarity and visual examples, others wanted clearer graphics and summary sections to facilitate rapid reference during time‐limited well‐baby visits:

There's not too much info. Everything's quite clear, not too much clicking. (P1_Family Medicine)

One thing I found a little more difficult … there's not a summary section or list. (P8_Family Medicine Urban, translated)

I really like the graphic. The pictures of the child, and what's typical and atypical, were very helpful. (P4_Family Medicine Rural)

There was a graphic of the child trying to reach for the ball. Could you put more examples of asymmetric postures or movements? Just so clinicians can visually see other cases. (P2_GP)

Is there another image? Could you put 2 images of a child being stiff? Is it just stiffness in the legs, or also the arms? (P3_Family Medicine Rural)

#### 3.2.4 Theme 4. Facilitators and Dissemination Strategies

The majority (*n* = 8/11, 73%) emphasized strategies to support widespread uptake, including embedding content into existing surveillance tools, residency curricula, nursing training and online platforms such as *About Kids Health* and professional societies. Posters, laminated cards and other visual aids were also highlighted as practical dissemination tools:

If there's a way to integrate it into residency programs … that would be super helpful. (P5_Family Medicine/Peds Urban)

Residency programs, especially family medicine. They don't get a lot of pediatric exposure. So the simple red flags would be great. (P6_GP)

We use About Kids Health a lot … it would be great if the tool could be linked there. (P4_Family Medicine Rural)

Of course, sending laminated copies that can be placed in the office would be very helpful. (P7_Nurse)

#### 3.2.5 Theme 5. Value and Impact for Practice

Seven participants (*n* = 7/11, 64%) described the **EDIT‐CP** toolkit, including **PROMPTs**, as educational and practical for structuring assessments, supporting decision‐making and guiding both new and experienced providers.

I thought the website was really helpful … I learned a couple of things too. (P3_Family Medicine Rural)

Several underscored the importance of making it usable across roles (family physicians, residents, nurse practitioners and nurse clinicians). They also underscored the value of involving parents in detection and follow‐up discussions:

It's good when parents are empowered with knowledge … if they don't see it, please bring it to your physician's attention. (P5_Family Medicine/Peds Urban)

#### 3.2.6 Theme 6. Barriers and Contextual Challenges

Six participants (55%) described barriers to implementation, especially in rural and remote contexts. They cited limited access to paediatric specialists, long delays for appointments and overall feasibility concerns. As one rural provider stated:

It's going to be extremely difficult for babies in our region to access these things because we're so remote. (P3_Family Medicine Rural)

Concerns extended beyond identification to the feasibility of accessing recommended interventions:

It just seems like it's gonna be very difficult to do any of these in our region. (P3_Family Medicine Rural)

Delays in accessing paediatric specialists and allied services were also described as significant barriers. A rural provider noted:

It's hard for babies to see a pediatrician, let alone like an acupuncture … even audiology appointments take months and months. (P3_Family Medicine Rural)

Similarly, variability in wait times across provinces was highlighted:

Sometimes seeing a pediatrician for these things takes 6 months, and then you refer out, and that takes another 6 months. (P6_GP)

Capacity constraints within specialist services were also raised, with one participant stating:

Some of these referrals that I sent to neurology end up being actually refused. (P6_GP)

Together, these findings suggest that while tools such as **PROMPTs** may enhance early recognition of motor concerns in primary care, timely access to specialist assessment and intervention remains highly dependent on local healthcare infrastructure.

### 3.3 Component 2—Paediatric Specialists—Perspectives on Referral Patterns and System‐Level Context

A focus group with developmental paediatricians (*n* = 7) and interviews with paediatric neurologists (*n* = 2) yielded six major themes regarding early CP detection and referral patterns.

#### 3.3.1 Theme 1. Referral Sources and Patterns

Eight participants (*n* = 8/9, 89%) noted that referrals now come from a broader range of providers, including family physicians, paediatricians, neonatologists and rehabilitation professionals. Several specialists described a shift toward more community‐based referrals compared to the past, when referrals were mostly between specialists. As one developmental paediatrician observed:

Referrals can come from family physicians, pediatricians, specialists … I've seen a really strong shift towards more and more questions around hypotonic cerebral palsy. (P3_Developmental Pediatrician)

Another added:

It used to be mostly from specialists to specialists … but we are now having more direct referrals from family physicians. (P6_Developmental Pediatrician)

Physiotherapists and proactive parents were often key triggers of earlier detection.

Parents are more aware with the Internet … they raise concerns, and physiotherapists often play a big role. (P1_Pediatric Physiatrist)

#### 3.3.2 Theme 2. Age of Referral and Diagnosis

Seven participants (*n* = 7/9, 78%) discussed progress in lowering the age of referral and diagnosis, particularly for infants at high probability of CP monitored in neonatal follow‐up programs. In these cases, tools such as the GMA and HINE were credited for facilitating diagnoses before 1 year of age. A paediatric neurologist explained:

We did see a decrease in the age of diagnosis, which is now 7 months for infants [at high‐probability for CP], compared to 24 months in our earlier study. (P9_Pediatric Neurologist)

Nonetheless, community referrals remain inconsistent and often late. As one developmental paediatrician noted:

I have seen a child 2 years and 6 months old as the youngest referred from the community. (P2_Developmental Pediatrician)

#### 3.3.3 Theme 3. Structural and Clinical Barriers to Early Diagnosis

Five participants (*n* = 5/9, 56%) highlighted persistent barriers to timely diagnosis. Several specialists described reluctance among physicians to diagnose CP before the age of two, even when clear signs were present. One developmental paediatrician summarized:

Physicians are still reluctant to diagnose CP under the age of 2. (P5_Developmental Pediatrician)

Another added:

There is hesitation … I can see that it's CP right away, but the neurologist does not want to put the diagnosis now. (P1_Developmental Pediatrician)

Equity concerns were also raised, with specialists noting that families able to access private physiotherapy often received earlier referrals and interventions compared to those dependent solely on the public system.

Parents … will quite often see a physiotherapy pediatric physiotherapist … and the physiotherapist will wake up and say, ‘Oh my God! There is an issue.’ … And I think that's why we see earlier patients. (P1_Pediatric Physiatrist)

In one interview, a paediatric neurologist similarly highlighted how some families are now accessing therapy in parallel to specialist assessment, expediting care:

It used to be that they would refer to pediatric neurology or developmental pediatrics, and they'd wait for us to then refer to rehab … Now it's much more parallel … I'm pleasantly pleased by how often these kids have been referred to physio already by the time they see me. So … it expedites the kids' assessment and intervention. (P9_Pediatric Neurologist)

Another developmental paediatrician emphasized inequities between infants at high probability of CP who are systematically followed and those from the community who are diagnosed later:

The ones [with high probability of CP] are followed very systematically … and they've always been diagnosed earlier and gotten interventions earlier, whereas those in the community tended to be diagnosed later and not get intervention. (P8_Developmental Pediatrician/Neurologist)

#### 3.3.4 Theme 4. Facilitators and System‐Level Enablers

Six participants (*n* = 6/9, 67%) emphasized system‐level strategies that could enable earlier detection. Embedding CP ‘red flag’ attributes into established surveillance frameworks such as the Rourke Baby Record and ABCDaire was viewed as especially promising. As one specialist explained:

Embedding the attributes into the Rourke record hits those immunization visits … that has the ability to drive change. (P5_Developmental Pediatrician)

Several also described the effectiveness of structured CME‐accredited training programs:

We developed CME‐accredited webinars and in‐person dinners across the province … it is very efficient. (P9_Pediatric Neurologist)

#### 3.3.5 Theme 5. Communication and Parental Engagement in Early Diagnosis

Six participants (*n* = 6/9, 67%) stressed the importance of communication strategies in supporting families. Specialists described the usefulness of adopting terms such as ‘high probability of CP’ to frame early concerns without alarming parents. As one developmental paediatrician put it:

Part of the teaching of the HINE is how to communicate … we've really been promoting the concept of “high probability of CP.” (P5_Developmental Pediatrician)

Participants noted that parents are increasingly proactive in raising concerns, often even before clinicians. One specialist remarked:

Parents are more aware with the Internet … they raise concerns, and physiotherapists often play a big role. (P1_Developmental Pediatrician)

#### 3.3.6 Theme 6. Dissemination Strategies

Seven participants (78%) highlighted dissemination approaches to strengthen uptake of early detection resources. These included leveraging professional societies, offering CME credits, embedding attributes into EMRs and targeting nurse practitioners. One paediatric neurologist suggested:

I would suggest CPS … and make sure CME [Continued Medical Education] credits are available. (P9_Pediatric Neurologist)

Another emphasized the role of nurse practitioners:

Nurse practitioners are becoming primary providers … this is a group that needs to be targeted. (P8_Pediatric Neurologist)

### 3.4 Merged Components

Across interviews and focus groups from both study components, several areas of convergence emerged between primary care providers and paediatric specialists (Table 2). Both groups emphasized the importance of clearer referral pathways, earlier recognition of CP and supportive, family‐centred communication. They also agreed on the strong educational and clinical value of early detection resources, highlighting the need to embed these attributes within existing surveillance tools, training programs and clinical workflows. Primary care providers focused on the practical usability of the resources, valuing bilingual access, visual clarity and quick navigation during well‐baby visits and emphasized the utility of posters, laminated cards and other visual aids to support point‐of‐care practice. Specialists, in contrast, underscored system‐level considerations, including persistent diagnostic hesitancy before age two, inequities in access to care and the need for structured CME‐accredited training, EMR integration and targeting of nurse practitioners as key dissemination strategies. Collectively, findings point to complementary priorities across the continuum of care: usability and accessibility in frontline practice, coupled with system‐wide education and policy supports to sustain early detection and timely referral.

**TABLE 2:** Themes and subthemes emerging from interviews and focus groups: Perspectives of primary care providers and paediatric specialists.

Theme	Primary care providers (Component 1, *n* = 11)	Specialists (Component 2, *n* = 9)	Convergence/divergence
Referral pathways and milestone interpretation	9 (82%) sought clearer referral pathways (paediatrician vs. neurologist), milestone refinement (corrected age and wider ranges) and more explanation on red flags (stiffness and asymmetry).	8 (89%) described increased referrals from primary care and earlier referrals for high‐probability infants via NICU follow‐up; community referrals still delayed and diagnostic hesitancy < 2 years persisted.	**Convergence:** Both emphasized the need for clearer referral guidance and earlier recognition. **Divergence:** Primary care focused on practical referral usability; specialists on diagnostic hesitancy and systemic delays.
Usability and integration of early detection resources	7 (64%) valued bilingual access, clear visuals and simple navigation; stressed the need to embed CP‐specific red flags into existing surveillance tools; requested more atypical posture examples and quick‐reference summaries.	6 (67%) emphasized embedding early detection attributes into existing surveillance tools (Rourke Baby Record, ABCDaire) and training curricula rather than specific website usability.	**Convergence:** Both valued accessible, structured resources and stressed the importance of embedding in existing surveillance tools. **Divergence:** Primary care emphasized visual usability; specialists emphasized system‐level and educational integration.
Dissemination and implementation strategies	8 (73%) recommended embedding content in residency/nursing programs, online platforms (About Kids Health, CPS and OIIQ) and practical visual aids (posters and laminated cards).	7 (78%) highlighted CME‐accredited sessions, webinars, EMR integration and targeting nurse practitioners.	**Convergence:** Education and embedding within existing structures. **Divergence:** Primary care valued tangible visual tools; specialists focused on CME and digital/system strategies.
Communication and parental involvement	6 (55%) described parents as key partners in identifying concerns, balancing empowerment and reassurance.	6 (67%) advocated for supportive communication using ‘high probability of CP’ terminology and noted parents' growing proactivity.	**Convergence:** Both valued parental involvement and supportive communication. **Divergence:** Specialists stressed diagnostic framing; primary care stressed avoiding parental fear.
Perceived value and clinical impact	7 (64%) viewed the toolkit and PROMPTs as educational and practical for structuring well‐baby assessments and clarifying roles.	6 (67%) regarded early detection tools as facilitating timely referrals and parallel access to rehabilitation.	**Convergence:** Both recognized strong educational and clinical value. **Divergence:** Primary care emphasized daily usability; specialists stressed accelerating access to intervention.
Barriers and contextual challenges	6 (55%) cited rural/remote barriers, limited specialist access and feasibility concerns.	4 (44%) described systemic inequities (public vs. private care), diagnostic hesitancy, and coordination gaps.	**Convergence:** Both identified access and feasibility barriers. **Divergence:** Primary care stressed geography; specialists emphasized systemic and equity issues.
Existing surveillance tools (Rourke Baby Record; ABCDaire)	7 (64%) compared both tools: ABCDaire favoured for usability, space and language accessibility; Rourke more complete but condensed.	6 (67%) referenced these as key embedding frameworks for early detection but did not compare usability.	**Convergence:** Both acknowledged their importance for integration. **Divergence:** Primary care compared usability; specialists focused on embedding early detection attributes.
Diagnostic hesitancy before age two	Not discussed	5 (56%) reported reluctance among clinicians to diagnose < 2 years despite clear signs.	**Unique to specialists:** Highlights ongoing need for confidence in early identification.

## 4 Discussion

This study explored primary care providers' perspectives on the feasibility, usability and integration of the **EDIT‐CP PROMPTs for referral** into routine surveillance and well‐baby visits, as well as strategies to support frontline adoption. It also examined specialists' perspectives on referral patterns, variability in age at CP diagnosis, and barriers to early identification to contextualize system‐level factors influencing dissemination and integration of early detection resources. Our findings reveal notable convergence across practitioner groups, significant alignment with previous evidence, and highlight the pressing need to bridge long‐standing diagnostic and implementation gaps. Broadly, both primary care clinicians and specialists in our study affirmed the value of embedding specific CP‐related red flags and warning signs into existing surveillance tools such as the Rourke Baby Record and ABCDaire. This aligns with implementation research showing that integration into established workflows significantly increases tool uptake (Maitre et al. 2020) and echoes international consensus urging that early detection strategies must be practical and system‐compatible (Novak et al. 2017; Hidalgo‐Robles et al. 2025).

Specialists in our study confirmed that neonatal follow‐up programs using the GMA and HINE have advanced early diagnosis in high‐probability infants, sometimes as early as 7 months corrected age. These findings are corroborated by retrospective and prospective studies demonstrating the high sensitivity and specificity of GMA and HINE when used in tandem (Morgan et al. 2019). Unfortunately, such resources for prompt identification is less achievable and feasible for infants outside NICU follow‐up, aligning with registry and survey data showing persistently delayed diagnosis, often beyond 24 months, for children with uneventful pregnancies and deliveries, with milder CP presentations (Boychuck et al. 2019; Boychuck, Andersen, Fehlings, et al. 2020; McIntosh et al. 2025). This gap highlights why tools like the **PROMPTs for referral** are essential in the primary care context, where most infants with mild or subtle presentations are first seen. Unlike tertiary settings, family physicians and community paediatricians rarely have access to GMA and/or HINE; instead, they rely on observable red flags, developmental surveillance and parental concerns during routine well‐baby visits. The **PROMPTs** provide concise, actionable indicators (e.g., persistent asymmetry, delayed head control or atypical postures) ready to be embedded into existing surveillance platforms (Rourke Baby Record and ABCDaire). By supporting nonspecialist providers to recognize early motor signs and initiate timely referrals, **PROMPTs** address the critical ‘blind spot’ in early CP detection: infants without classic risk factors (e.g., born preterm and neonatal encephalopathy), who represent the majority of the CP population. In this way, **PROMPTs** complement specialized tools like GMA and HINE by extending early identification capacity into frontline practice, where the largest opportunity exists to reduce inequities in age of diagnosis.

Furthermore, clinician uncertainty and a long‐standing professional norm of deferring definitive CP diagnosis until after age two emerged as key barriers to early action. These patterns are consistent with studies across Canada and internationally that identify gaps in diagnostic confidence, variability in knowledge and limited structured referral pathways among community paediatricians. Indeed, a bc‐based initiative developed a diagnostic care pathway precisely to address such gaps (Scoten et al. 2025). This reluctance is not unique to Canada: surveys of physicians in the United States, Australia and Europe have similarly reported that many providers prefer to use terms such as ‘motor delay’ or ‘developmental concern’ rather than label a child with CP probability before the second birthday, even when diagnostic criteria are met (Novak et al. 2017) (Kim et al. 2024). Such hesitancy is often fuelled by concerns about misdiagnosis, fear of alarming families or uncertainty regarding prognosis in milder cases (Scherzer et al. 2012). However, evidence indicates that delaying a diagnosis not only postpones access to critical early interventions but also undermines caregiver trust, as many parents report feeling dismissed or left in limbo during this period of uncertainty (Williams et al. 2021; Baird et al. 2000).

Importantly, emerging consensus guidelines emphasize that an early CP diagnosis should be made when sufficient clinical evidence exists, even if functional severity is not yet fully defined, with the option of framing the diagnosis as ‘high probability of CP’ (Novak et al. 2017; Aravamuthan et al. 2025). This approach has been shown to reduce diagnostic delays while still allowing clinicians to acknowledge uncertainty and communicate ongoing developmental surveillance. Our findings suggest that without explicit pathways and tools, such as **PROMPTs for referral**, many frontline providers may default to a ‘wait and see’ approach, inadvertently prolonging the time before referral and intervention. This pattern is consistent with prior work: Tang et al. (2012) showed that even in neonatal follow‐up programs where developmental concerns were identified, less than one‐third of infants were actually referred for early intervention, illustrating how recognition alone does not translate into action (Tang et al. 2012). Likewise, a recent systematic review demonstrated that multicomponent referral strategies, which combine explicit algorithms, workflow integration and provider education, were more effective than isolated tools in improving referral practices (Bolton Saghdaoui et al. 2025). Together, these findings reinforce the importance of structured tools like **PROMPTs** to bridge the gap between early recognition and timely referral.

Communication surfaced as a critical domain in the diagnostic process. Clinicians highlighted the importance of using neutral, carefully chosen language, such as ‘high probability of CP’, to convey diagnostic certainty while avoiding unnecessary alarm. This approach aligns with caregiver‐centred frameworks, which emphasize that parents value early and honest communication when framed with empathy, transparency and clear next steps for action (Williams et al. 2021; Kim et al. 2025). Ambiguity or delayed disclosure has previously been shown to heighten parental anxiety and erode trust in the healthcare system (Baird et al. 2000; Dagenais et al. 2006). Best practice guidelines therefore recommend that CP diagnoses, or provisional designations, be delivered not as a one‐time event but as a process: across multiple conversations, in plain language, using balanced framing of both challenges and strengths and supplemented with written resources and structured follow‐up supports (Novak et al. 2017).

Importantly, communication must also account for cultural and linguistic diversity. Families from different cultural backgrounds vary in their expectations regarding diagnostic disclosure, tolerance for uncertainty and involvement of extended family. A recent qualitative study reported that while many caregivers accept early conversations about CP (or a high‐probability designation), they often feel unprepared and report emotional distress when information is poorly framed (Kim et al. 2025). Language barriers compound these challenges. For instance, hospitalized children of parents with limited English proficiency experience more than double the risk of adverse events compared to those whose parents are comfortable with English (Khan et al. 2020), and broader reviews confirm language discordance undermines care quality and patient satisfaction (The impact of language barriers on quality and safety in health care 2018). Culturally responsive approaches, such as professional interpretation and translated, visually aided materials, are therefore essential. Our findings affirm that how we communicate (not just when) is critical for equity and family‐centred care.

Implementation and dissemination strategies identified by participants, such as embedding tools into EMRs, providing CME‐accredited training, engaging nurse practitioners and using visual aids, reflect core implementation science principles. The Consolidated Framework for Implementation Research (CFIR) highlights how constructs such as intervention characteristics, clinician capability, organizational readiness and external context shape adoption and sustainability (Ardito et al. 2023; Rangachari et al. 2022). These findings parallel evidence from other healthcare innovations, where integrating new tools into existing workflows and ensuring leadership engagement were critical for uptake. A comparable example comes from the implementation of early intensive manual therapies (constraint‐induced movement therapy and bimanual training) for children with CP. Despite strong evidence supporting their clinical benefits, Canadian programs reported barriers including limited resources, insufficient training and systemic constraints, factors that align closely with CFIR domains (Vurrabindi et al. 2025). This underscores that even when interventions are evidence‐based and widely endorsed, successful implementation requires coordinated, multilevel strategies. Importantly, we have already designed and implemented training on the **EDIT‐CP** intervention component, which demonstrated positive effects on clinician knowledge and practice (Hanson et al. 2026), providing preliminary evidence that structured, targeted training can facilitate adoption. This underscores that even when interventions are evidence‐based and widely endorsed, successful implementation requires coordinated, multilevel strategies. Similarly, the **PROMPTs** and **EDIT‐CP** toolkit, though recognized as clinically valuable by both primary care providers and specialists, will require training, workflow integration and supportive policy measures to achieve consistent and equitable uptake across Canada.

### 4.1 Study Limitations

Several limitations in the present study warrant acknowledgment. Although this study provides important national insights, its generalizability may be influenced by the recruitment profile. Participation was primarily drawn from tertiary academic centres and therefore may not fully reflect the perspectives of clinicians and families practising in rural or remote regions. Access to specialized paediatric services varies considerably across Canada, and service delivery realities outside urban centres may differ substantially. In addition, although the study aimed to capture broad national representation, engagement with First Nations, Inuit and Métis communities was not achieved in this survey cycle. Given the importance of culturally safe and geographically accessible models of care, future research should prioritize intentional partnership with Indigenous communities and increased inclusion of rural professionals. A subsequent survey cycle or focused qualitative inquiry centred on these stakeholders would strengthen the development of a more inclusive and nationally representative strategy for CP diagnosis and management. Furthermore, the voluntary recruitment likely favoured individuals with pre‐existing interest or familiarity with early detection of CP, potentially inflating endorsement of the tools. Further, caregiver perspectives were outside the scope of this study but remain critical to understanding acceptability, communication preferences and family engagement.

Future research must focus on pragmatic implementation trials evaluating EMR‐embedded **PROMPTs** across diverse settings, measuring actual changes in referral timing and intervention access. Codesign with caregivers and allied health professionals is essential to ensure tools are culturally appropriate, accessible and aligned with parental needs. Mixed method approaches that include quantitative metrics (e.g., change in diagnostic age) and qualitative insights (e.g., family experience) will be invaluable.

## 5 Conclusion

This study identified alignment between primary care providers and paediatric specialists on the need for clearer referral pathways, earlier recognition of CP and practical tools to support decision‐making. Primary care clinicians viewed the **PROMPTs** as feasible and educational, particularly when integrated into existing surveillance tools and workflows. Specialists highlighted ongoing variability in referral timing, diagnostic hesitancy before age 2 and inequities in access to services, underscoring system‐level factors that influence early identification. Together, these findings suggest that structured referral guidance, embedded within routine practice and supported by targeted education and dissemination strategies, may help bridge gaps between early recognition and timely referral across the continuum of care.

## Author Contributions

**Darcy Fehlings:** conceptualization, investigation, funding acquisition, methodology, writing – review and editing, supervision. **Annette Majnemer:** conceptualization, investigation, funding acquisition, methodology, writing – review and editing, supervision. **Mae Ru Sanford:** visualization, formal analysis. **Martina Alkot:** visualization, formal analysis, writing – original draft. **Tatiana Ogourtsova:** writing – original draft, funding acquisition, investigation, conceptualization, visualization, writing – review and editing, formal analysis, supervision.

## Funding

This study was supported by the Kids Brain Health Network.

## Disclosure

ChatGPT (OpenAI) was used to assist with language editing and improving clarity during manuscript preparation. The authors reviewed and edited all of the content and take full responsibility for the final manuscript.

## Ethics Statement

Ethics approval for this study was obtained from the Research Institute of the McGill University Health Center review board (Approval No. MP‐37‐2023‐8827, Date of Approval: 2023‐04‐20). All study procedures complied with institutional and national guidelines for research involving human participants.

## Consent

All participants provided informed consent prior to participation.

## Conflicts of Interest

The authors declare no conflicts of interest.

## Supporting information


**Data S1:** Preinterview materials and focus group questions.

## Data Availability

The qualitative data supporting the findings of this study are not publicly available due to confidentiality agreements with participants and restrictions related to protecting individual privacy. Deidentified excerpts may be available from the corresponding author upon reasonable request and subject to institutional ethics approval.
