# Supplementary material for: Bridging the Gap in Early Cerebral Palsy Detection: Primary Care Providers' and Specialists' Perspectives on Implementing PROMPTs for Referral
Source: Child Care Health Dev. 2026 May 10;52:e70287. doi: 10.1111/cch.70287 (PMC13158324; doi:10.1111/cch.70287)
Supplement: Supplementary file 1 — Data S1: Preinterview materials and focus group questions. [file CCH-52-e70287-s001.docx]

**Supplemnetary file 1:** Pre-interview materials and focus group questions

**Early Detection and Intervention Toolkit for Children with Cerebral Palsy (EDIT-CP): A National-Level Knowledge Implementation Project**

**Pre-Interview Materials (primary care)**

Thank you for agreeing to take part in this research study about the early identification of young children not yet diagnosed with cerebral palsy (CP). Our team has developed internationally validated knowledge translation tools to assist primary care providers (family physicians, community-based pediatricians) in the early detection of cerebral palsy, with recommendations for referral to appropriate specialists. This feedback collected from primary care providers such as yourself will help our research team optimize these tools for implementation nationally.

To participate in this interview, you will need to complete and sign the attached consent form. Once that is received, we will reach out to you to schedule a mutually convenient time for a 30-45 minute interview to be conducted via Zoom. We are also including a list of interview questions below to help you optimally prepare for the interview. If you have any questions at any point, please reach out to the study coordinator xx.

**► Interview Questions Pertaining to the Rourke Baby Record:** <https://www.rourkebabyrecord.ca/rbr2020/default>

Prior to the interview, we ask that you please look at the relevant items on the Rourke Baby Record to assess both the feasibility and likelihood of applying the attributes as presented to your well-baby care routine for infants seen in the first year of life.

1. How feasible is it to apply the attributes as presented in the Rourke Baby Record to your well-baby care routine for infants seen in the first year of life?

- Are the attributes easy to access when needed?
- Are the attributes clear and well-presented/explained?
- Are the attributes easy to understand?
- Are you satisfied with these attributes as outlined?

1. How likely are you to apply the attributes as presented in the Rourke Baby Record to your well-baby care routine for infants seen in the first year of life?

- What do you see as potential barriers to applying these attributes that could limit your use of it as part of your practice?
- What would facilitate your application of these attributes?
- What do you see as the best ways to integrate this tool into frontline primary care?
- How would your practice change by using this tool?

**► Interview Questions Pertaining to the EDIT-CP Website:** <https://www.childhooddisability.ca/early-detection-of-cp/>

Prior to the interview, we ask that you please look at our bilingual web-based tools to assess both the feasibility and likelihood of using this resource (appropriateness) as part of your well-baby care routine for infants seen in the first year of life.

1. How feasible is it to use this website as part of your well-baby care routine for infants seen in the first year of life?

- Is the website easy to access and navigate?
- Are the different sections of the website, including the graphics, clear and well-presented/explained?
- Is the website content easy to understand?
- Are you satisfied with this website as presented?

1. How likely are you to use this website as part of your well-baby care routine for infants seen in the first year of life?

- What do you see as potential barriers to using this website that could limit your use of it as part of your practice?
- What would facilitate your use of this website?
- What do you see as the best ways to integrate use of this website into frontline primary care?
- How would your practice change by using this website?

**Focus group questions (specialists)**

**Introduction (Context Provided to Participants)**

Participants were informed that the purpose of the interview was to explore whether there have been changes in referral and diagnostic patterns for CP following dissemination of the EDIT-CP toolkit and PROMPTs for referral, particularly among children not enrolled in neonatal follow-up programs. The toolkit aims to support earlier identification and referral in community settings.

**1. Professional Role and Clinical Context**

1. What is your current position and clinical role?
2. What proportion of children with CP in your practice come from:
   - a) High-risk neonatal follow-up populations?
   - b) Community referrals (children not followed in NICU programs)?

**2. Changes in Referral Patterns**

1. Have you observed changes in referral patterns over the past 1–2 years, particularly for children not followed in neonatal programs?
   - Specifically, have you noticed changes in:
     - Age at referral?
     - Clinical signs prompting referral?
2. Have you observed changes in referral sources?
   - For example, referrals from:
     - Primary care physicians
     - Community pediatricians
     - Other providers
3. When did you begin noticing any change (if applicable)?

**3. Referral to Rehabilitation Services**

1. Are children being referred to rehabilitation services (e.g., physiotherapy, occupational therapy) prior to specialist assessment?
2. Where are these referrals typically directed (e.g., hospital-based services, rehabilitation centers, community programs)?

**4. Dissemination and Implementation**

1. Do you have recommendations for additional strategies to disseminate early identification information to community providers?
   - Are there specific provider groups that should be targeted?
2. What barriers do you perceive to effective dissemination and uptake of early identification tools?

**5. Closing**

1. Is there anything else you would like to add regarding referral patterns, early diagnosis, or implementation of the toolkit?
